# Rheumatoid Arthritis and Thrombocytopenia: Causal Evidence and Prognostic Implications From Observational and Mendelian Randomization Analyzes

**DOI:** 10.1002/iid3.70551

**Published:** 2026-11-11

**Authors:** Yejun Wu, Yuyang Cai, Anzhen Yang, Shuying Zhang, Liangyu Xu, Yanting Xie, Peng Zhao

**Affiliations:** ^1^ School of Clinical Medicine Shanghai University of Medicine and Health Sciences Shanghai China; ^2^ Pudong Gongli Hospital Shanghai University of Medicine and Health Sciences Shanghai China; ^3^ Peking University People's Hospital, Peking University Institute of Hematology Beijing China

**Keywords:** all‐cause mortality, autoimmunity, Mendelian randomization, NHANES, rheumatoid arthritis, thrombocytopenia

## Abstract

**Background:**

Rheumatoid arthritis (RA) is a systemic autoimmune disease associated with hematological abnormalities, and we aimed to investigate the clinical significance and causal relationship of thrombocytopenia (TP) in RA. We integrated observational data with genetic approaches, characterized the clinical features of RA patients with TP, and evaluated their prognostic implications.

**Materials and Methods:**

Data from the National Health and Nutrition Examination Survey were used to analyze clinical characteristics and survival outcomes of RA patients. Survey‐weighted Cox regression models were applied to evaluate the association between TP and mortality. Two‐sample Mendelian randomization (MR) analysis was performed to evaluate the causal relationship between RA and TP.

**Results:**

Among 2667 RA participants with available platelet measurements, 135 had TP (< 150 × 10^9^/L). In the common complete‐case sample, TP remained associated with higher all‐cause mortality after full survey‐weighted adjustment (HR 1.92, 95% CI 1.17–3.16; *p* = 0.009). Lower serum albumin and higher urinary albumin concentration were also associated with mortality in the fully adjusted model. In the MR analysis using 52 SNPs, fixed‐effect IVW yielded OR 1.054 (95% CI 1.002–1.108; *p* = 0.042), but alternative estimators were not significant and the MR‐Egger intercept indicated directional pleiotropy (*p* = 0.0016).

**Discussion:**

Thrombocytopenia was associated with poorer survival among RA participants, whereas MR did not provide robust evidence for a causal effect of RA liability on thrombocytopenia. These findings warrant further prospective validation.

**Abbreviations**
BMI: body mass index
CDC: Centers for Disease Control and Prevention
CI: confidence interval
CRP: C‐reactive protein
DBP: diastolic blood pressure
GGT: gamma‐glutamyl transferase
GWAS: genome‐wide association study
HDL: high‐density lipoprotein
HR: hazard ratio
hsCRP: high‐sensitivity C‐reactive protein
ITP: immune thrombocytopenia
IVW: inverse variance weighted
LD: linkage disequilibrium
MR: Mendelian randomization
NHANES: National Health and Nutrition Examination Survey
OR: odds ratio
RA: rheumatoid arthritis
SBP: systolic blood pressure
SD: standard deviation
SNP: single nucleotide polymorphism
TP: thrombocytopenia

## 1 Introduction

Rheumatoid arthritis (RA) is an immune‐mediated disorder in which chronic synovitis progressively damages joints [1, 2]. Its clinical burden, however, is not confined to the musculoskeletal system: extra‐articular involvement and hematologic abnormalities may complicate disease management [3, 4, 5, 6]. Reduced platelet counts are of particular concern because they can increase bleeding liability and may constrain treatment choices [7, 8].

Thrombocytopenia (TP) can result from reduced platelet production, increased destruction or consumption, sequestration, or immune‐mediated clearance [7, 8]. In general clinical practice, TP is commonly defined as a platelet count < 150 × 10^9^/L, whereas < 100 × 10^9^/L is used in international definitions of immune thrombocytopenia (ITP) [7, 9, 10]. Recent population‐based work has likewise distinguished broad TP (< 150 × 10^9^/L) from ITP (< 100 × 10^9^/L) with ICD‐10 coding [11]. In RA, TP may be autoimmune, drug‐related, or secondary to comorbid conditions, and available RA‐specific epidemiologic evidence remains limited [5, 6, 12, 13]. However, the prognostic significance of TP in RA remains uncertain.

RA is associated with excess all‐cause and cardiovascular mortality compared with the general population, underscoring the importance of identifying clinically vulnerable subgroups [14, 15, 16, 17]. We therefore combined genetic and population‐based approaches. Two‐sample Mendelian randomization (MR) was used to test whether inherited susceptibility to RA is related to TP, while NHANES data were used to characterize RA participants with TP and their subsequent survival. Exploratory SMR analyzes were additionally used to assess selected JAK‐related inflammatory and downstream signaling targets.

## 2 Methods

### 2.1 Study Framework

The project comprised three linked components: an NHANES analysis of clinical characteristics and mortality in RA, a two‐sample MR analysis of RA liability and thrombocytopenia, and an exploratory eQTL‐based assessment of selected JAK‐related targets.

### 2.2 Data Sources

Observational data were obtained from 10 continuous National Health and Nutrition Examination Survey (NHANES) cycles conducted between 1999 and 2018. NHANES uses a complex, multistage probability sampling design and combines interview data with standardized examinations and laboratory measurements conducted at mobile examination centers. The present analysis incorporated examination sampling weights, strata, and primary sampling units to account for the complex survey design [18]. RA GWAS summary statistics were obtained from the European‐ancestry GWAS reported by Ishigaki et al. (2022), available from the NHGRI‐EBI GWAS Catalog under accession GCST90132223 [19]. This dataset included 22350 RA cases and 74823 controls of European ancestry. The thrombocytopenia outcome was obtained from FinnGen Release 12, endpoint D3_THROMBOCYTOPENIANAS, corresponding to ICD‐10 D69.6 (thrombocytopenia, unspecified) [20]. Cis‐eQTL summary data used for the exploratory SMR analyzes were obtained from the eQTLGen Consortium [21].

### 2.3 NHANES Cohort and Outcome Definitions

Participants were selected from the 1999 to 2018 NHANES cycles. The initial combined dataset included 101316 participants. To preserve the NHANES examination‐based complex survey design, participants with positive MEC examination weights were first identified (*n* = 96766), and the survey design was constructed using the MEC examination weight, sampling strata, and primary sampling units. Participants with available platelet measurements were then identified (*n* = 82926), and analyzes of rheumatoid arthritis were conducted as survey‐domain analyzes.

RA status was identified from the arthritis questionnaire and the recorded arthritis subtype. The final baseline RA cohort consisted of 2667 participants with available platelet counts. TP was primarily defined as a platelet count < 150 × 10^9^/L, yielding 135 participants with TP and 2532 without TP. A stricter threshold of < 100 × 10^9^/L was examined in sensitivity analyzes [7, 9, 10, 11].

Mortality status was obtained from the NHANES Linked Mortality File. Among the baseline RA cohort, 2664 participants had valid mortality follow‐up information and were included in the survey‐weighted survival analysis. Sequential Cox regression analyzes were performed in a common complete‐case sample of 1963 participants to ensure an identical analytical population across progressively adjusted models.

### 2.4 Covariates and Mortality Ascertainment

Variables considered in descriptive or regression analyzes included demographic characteristics (age, sex, race/ethnicity, education, and marital status), anthropometric measures, blood pressure, platelet count, serum uric acid, serum albumin, urinary albumin concentration, gamma‐glutamyl transferase, and high‐sensitivity C‐reactive protein.

Where necessary, skewed laboratory measures were transformed. Variables with substantial cycle‐specific missingness, including serum ferritin, plasma fibrinogen, and high‐sensitivity C‐reactive protein, were not retained in the revised main analyzes. No missing‐data imputation was performed.

Vital status was obtained from the NHANES Linked Mortality File/National Death Index linkage. Person‐time was measured from the NHANES interview date through death or December 31, 2019, whichever came first.

For the survival component, all‐cause mortality was the endpoint. Participants were followed until death or administrative censoring on December 31, 2019. Prescription‐medication data were obtained from the NHANES prescription‐medication files, which record medications used during the preceding month [22]. Given their established role in rheumatoid arthritis management, indicators of glucocorticoid use and conventional synthetic disease‐modifying antirheumatic drug (csDMARD) use were derived and included in medication‐adjusted sensitivity analyzes [23]. Medication variables were used to assess potential confounding rather than to estimate causal treatment effects.

### 2.5 Mendelian Randomization and Target‐Gene Analyzes

MR estimates were interpreted under the usual instrumental‐variable requirements: selected variants must predict RA, be unrelated to relevant confounders, and affect TP through RA rather than an alternative pathway [24, 25]. Exploratory SMR analyzes used cis‐eQTL summary statistics from the eQTLGen Consortium [21]. RA‐associated variants reaching *P* < 5 × 10^−8^ were clumped using a 10,000‐kb window and r^2^ < 0.001 with the 1000 Genomes European reference panel. After outcome matching and allele harmonization, 52 SNPs were retained. Instrument strength was assessed using F = (β/SE)^2^; all retained instruments had F > 10 [26] (Supporting Information Table S3). Study‐specific effect‐allele frequencies were unavailable; therefore, SNP‐specific R^2^ was not calculated.

After allele alignment with the FinnGen TP dataset, inverse‐variance weighting (IVW) served as the principal estimator. MR‐Egger and weighted‐median estimates were generated as robustness checks [27, 28], together with weighted‐mode and simple‐mode estimates. Cochran's Q quantified between‐SNP heterogeneity, and the MR‐Egger intercept was used to screen for directional pleiotropy [27]. Leave‐one‐out analysis and MR‐PRESSO were additionally performed as sensitivity analyzes [29].

For JAK1 and JAK2, stringent cis‐eQTL filtering left too few independent instruments for a stable conventional multi‐instrument MR analysis. We therefore used SMR with HEIDI testing [30]. In this analysis, SMR assesses the gene‐expression/outcome signal, whereas HEIDI evaluates whether that signal is compatible with a shared variant rather than linkage. To address JAK‐related upstream and downstream targets, exploratory analyzes were extended to IFNG, IL1B, TNFRSF1A, TNFRSF1B, IL6, JAK1, JAK2, STAT1, STAT3, STAT5A, and STAT5B. D69.6 was used as the principal thrombocytopenia outcome, with D69.5 examined additionally, and multiple‐testing correction was applied across the target panel. Two‐sample MR analyzes were performed in R 4.5.2 using TwoSampleMR [31]; SMR/HEIDI analyzes used SMR v1.3.2. An SMR *p* value < 0.05 was treated as nominal evidence, and a HEIDI *p* value < 0.01 was considered evidence of heterogeneity.

### 2.6 Statistical Analysis

Baseline values were compared between TP and non‐TP groups using the NHANES complex survey design. Design‐based Rao–Scott tests were used for categorical variables and survey‐weighted t tests for continuous measures. Survival functions were estimated using survey‐weighted methods and compared using a design‐based log‐rank test.

A series of sequential survey‐weighted Cox proportional hazards models was fitted to evaluate the association between thrombocytopenia and all‐cause mortality. Model 1 was unadjusted. Model 2 adjusted for age, sex, and race/ethnicity. Model 3 additionally adjusted for education, marital status, poverty‐income ratio, smoking status, and body mass index. Model 4 further adjusted for systolic blood pressure, diastolic blood pressure, serum uric acid, serum albumin, natural log‐transformed urinary albumin concentration, and gamma‐glutamyl transferase. All four models were fitted using the same common complete‐case sample to ensure that differences in effect estimates reflected progressive covariate adjustment rather than differences in the analytical population. Results are reported as hazard ratios (HRs) with 95% confidence intervals (CIs). All tests were two‐sided; *p* < 0.05 defined statistical significance.

Sensitivity analyzes included an unweighted Cox model on the same complete‐case sample, a stricter platelet threshold (< 100 × 10^9^/L), additional adjustment for glucocorticoid and csDMARD use, alternative examination‐based follow‐up time, and exclusion of participants with current liver conditions and/or hematologic malignancy history. The proportional hazards assumption was evaluated using Schoenfeld‐residual diagnostics in auxiliary Cox models. When a covariate showed evidence of possible non‐proportionality, a time‐varying coefficient model was additionally fitted.

## 3 Results

### 3.1 Baseline Profile

Among 2667 participants with RA and available platelet measurements, 135 had platelet counts < 150 × 10^9^/L and 2532 had platelet counts ≥ 150 × 10^9^/L (Table 1). Participant selection is summarized in Figure 1. Baseline characteristics were estimated using the NHANES complex survey design.

**Table 1:** Clinical characteristics of rheumatoid arthritis patients stratified by thrombocytopenia status (platelet count < 150 × 109/L).

Characteristics	RA patients		*p* value
	Without TP (*n* = 2532)	With TP (*n* = 135)	
**Age group, *n* (%)**			0.013
< 60 years	1115 (53.8%)	46 (40.0%)	
≥ 60 years	1417 (46.2%)	89 (60.0%)	
Male sex, *n* (%)	1010 (40.3%)	82 (55.0%)	0.013
**Race/ethnicity, *n* (%)**			0.325
Mexican American	411 (6.5%)	23 (7.5%)	
Other Hispanic	205 (5.1%)	7 (4.3%)	
Non‐Hispanic White	1056 (66.8%)	51 (60.3%)	
Non‐Hispanic Black	727 (16.1%)	47 (23.2%)	
Other Race	133 (5.5%)	7 (4.7%)	
**Education, *n* (%)**			0.528
College graduate or above	288 (14.4%)	18 (11.8%)	
High school graduate/GED or some college/AA degree	1309 (60.7%)	69 (58.6%)	
Less than high school degree	871 (25.0%)	44 (29.6%)	
**Marital status, *n* (%)**			0.399
Married/living with partner	1396 (63.0%)	72 (56.0%)	
Widowed/divorced/separated	865 (29.8%)	49 (36.1%)	
Never married	215 (7.2%)	12 (7.9%)	
**Smoking status, *n* (%)**			0.616
Never smoker	1140 (41.8%)	55 (38.1%)	
Former smoker	799 (31.6%)	43 (37.0%)	
Current smoker	590 (26.5%)	37 (24.9%)	
BMI, kg/m^2^	30.30 (0.22)	29.13 (0.58)	0.062
Serum uric acid, mg/dL	5.59 (0.04)	5.66 (0.24)	0.791
Serum albumin, g/dL	4.16 (0.01)	4.02 (0.04)	< 0.001
Urinary albumin concentration, natural log mg/L	2.30 (0.04)	2.66 (0.15)	0.020
GGT, U/L	33.49 (1.05)	70.06 (13.34)	0.007
Platelet count, ×10^9^/L	262.10 (1.86)	123.69 (2.55)	< 0.001

*Note:* Values are unweighted n (survey‐weighted %) for categorical variables and survey‐weighted mean (SE) for continuous variables. Design‐based Rao–Scott tests were used for categorical variables and survey‐weighted tests for continuous variables.

Abbreviations: BMI, body mass index; GGT, gamma‐glutamyl transferase; RA, rheumatoid arthritis; TP, thrombocytopenia.

**Figure 1:**
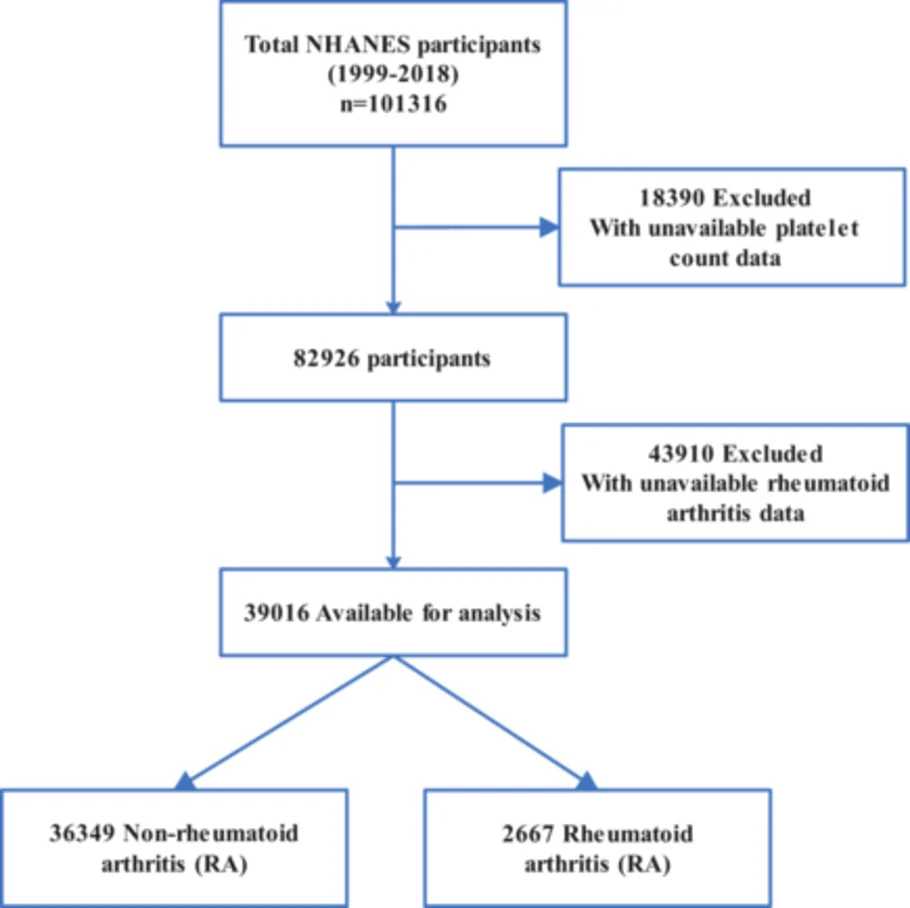
Participant‐selection flowchart for the NHANES 1999–2018 analysis.

Participants with TP were more frequently male and aged ≥ 60 years. Serum albumin was lower, whereas urinary albumin concentration and gamma‐glutamyl transferase were higher in the thrombocytopenia group. Detailed survey‐weighted baseline characteristics are presented in Table 1.

### 3.2 Survival in RA

Among 2664 RA participants with valid mortality follow‐up, 805 deaths occurred. Survey‐weighted survival was lower among participants with platelet counts < 150 × 10^9^/L than among those with platelet counts ≥ 150 × 10^9^/L (design‐based *p* = 0.001; Figure 2).

**Figure 2:**
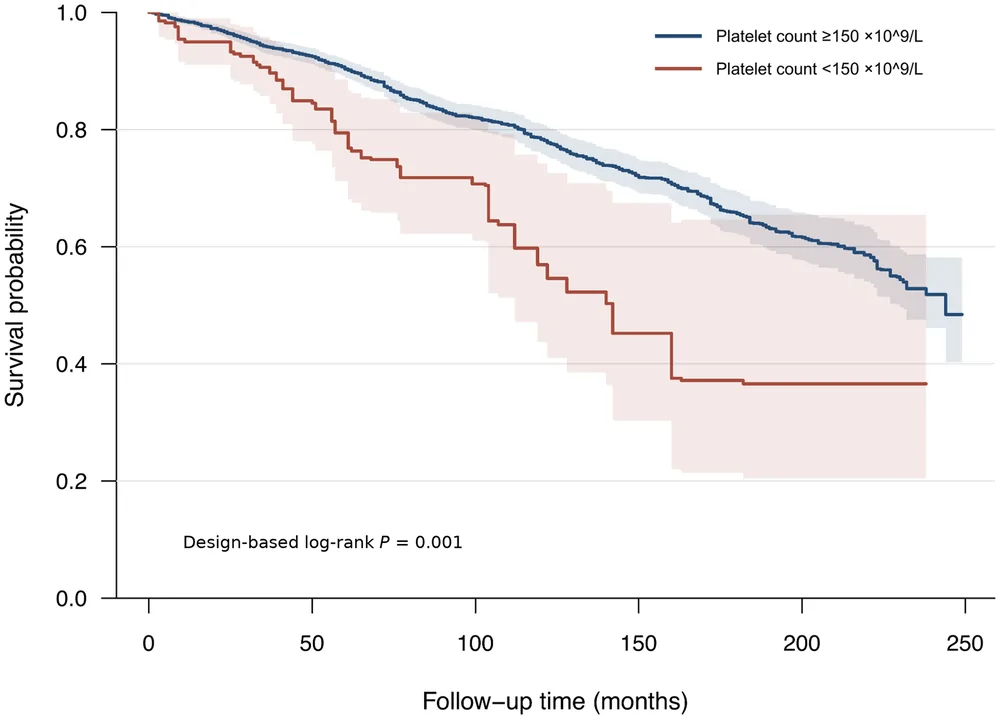
Survey‐weighted survival curves for rheumatoid arthritis participants with and without thrombocytopenia using the 150 × 10^9^/L threshold. Shaded areas indicate 95% confidence intervals; the design‐based log‐rank *p* value was 0.001.

Sequential Cox analyzes were performed in a common complete‐case sample of 1963 participants, including 529 deaths. Thrombocytopenia was consistently associated with higher all‐cause mortality across increasing levels of adjustment. The HR was 2.55 (95% CI 1.46–4.45; *p* = 0.001) in Model 1, 2.40 (95% CI 1.44–4.03; *p* < 0.001) in Model 2, 2.32 (95% CI 1.37–3.96; *p* = 0.002) in Model 3, and 1.92 (95% CI 1.17–3.16; *p* = 0.009) in the fully adjusted Model 4 (Table 2).

**Table 2:** Survey‐weighted Cox regression analysis of the association between thrombocytopenia and all‐cause mortality in rheumatoid arthritis patients.

Model	Adjustments	HR (95% CI)	*p* value
Model 1	Unadjusted.	2.55 (1.46–4.45)	0.001
Model 2	Adjusted for age, sex, and race/ethnicity.	2.40 (1.44–4.03)	< 0.001
Model 3	Model 2 + education, marital status, poverty‐income ratio, smoking status, and BMI.	2.32 (1.37–3.96)	0.002
Model 4	Model 3 + systolic blood pressure, diastolic blood pressure, serum uric acid, serum albumin, natural log urinary albumin concentration, and GGT.	1.92 (1.17–3.16)	0.009

*Note:* All models were fitted using the same common complete‐case sample (*N* = 1963; deaths = 529). Model 1 was unadjusted; Models 2–4 used the sequential adjustments described in Methods. TP was defined as platelet count < 150 × 10^9^/L.

Abbreviations: CI, confidence interval; HR, hazard ratio.

In Model 4, higher systolic blood pressure, serum uric acid, and natural log‐transformed urinary albumin concentration were associated with higher mortality, whereas higher diastolic blood pressure and serum albumin were associated with lower mortality. Gamma‐glutamyl transferase was not significantly associated with mortality (Table 3).

**Table 3:** Clinical and laboratory factors in the fully adjusted Cox regression model for all‐cause mortality in rheumatoid arthritis patients.

Variable	Level or Unit	HR (95% CI)	*p* value
Systolic blood pressure	Per 1 mmHg increase	1.0100 (1.0044–1.0157)	< 0.001
Diastolic blood pressure	Per 1 mmHg increase	0.9848 (0.9795–0.9902)	< 0.001
Serum uric acid	Per 1 mg/dL increase	1.23 (1.14–1.32)	< 0.001
Serum albumin	Per 1 g/dL increase	0.33 (0.23–0.45)	< 0.001
Urinary albumin concentration (log)	Per 1 natural‐log unit increase	1.111 (1.008–1.225)	0.034
GGT	Per 1 U/L increase	1.0007 (0.9998–1.0016)	0.133

*Note:* Model 4 additionally adjusted for thrombocytopenia, age, sex, race/ethnicity, education, marital status, poverty‐income ratio, smoking status, and BMI.

Abbreviations: CI, confidence interval; GGT, gamma‐glutamyl transferase; HR, hazard ratio.

### 3.3 Sensitivity Analyzes

Additional adjustment for prescription medication use did not materially alter the association between thrombocytopenia and mortality. In the medication‐complete sample (*n* = 1929), the Model 4 estimate was HR 1.92 (95% CI 1.17–3.16; *p* = 0.010) before medication adjustment and HR 1.91 (95% CI 1.17–3.14; *p* = 0.010) after additional adjustment for glucocorticoid and csDMARD use (Supporting Information Table S1).

Global Schoenfeld‐residual tests did not indicate violation of the proportional hazards assumption in Model 3 (*p* = 0.521) or Model 4 (*p* = 0.170), and no evidence of non‐proportionality was observed for thrombocytopenia itself. Although possible time‐varying effects were identified for BMI in Model 3 and log‐transformed urinary albumin concentration in Model 4, corresponding time‐varying coefficient analyzes produced nearly unchanged thrombocytopenia estimates (Supporting Information Table S2).

Additional sensitivity analyzes were directionally consistent with the primary result: the unweighted Model 4 yielded HR 1.69 (95% CI 1.16–2.47; *p* = 0.006), the < 100 × 10^9^/L threshold yielded HR 3.33 (95% CI 1.20–9.22; *p* = 0.021), and examination‐based follow‐up yielded HR 1.94 (95% CI 1.18–3.19; *p* = 0.009). Exclusion of hematologic malignancy had little effect (HR 1.94, 95% CI 1.17–3.21; *p* = 0.010), whereas exclusion of participants with a current liver condition attenuated the association (HR 1.48, 95% CI 0.87–2.55; *p* = 0.151) (Supporting Information Table S1).

### 3.4 MR Findings

After clumping, outcome matching, and harmonization, 52 independent RA‐associated SNPs were retained; all had F statistics > 10 (minimum 29.70, Supporting Information Table S3). Fixed‐effect IVW showed a nominal association between RA genetic liability and D69.6 thrombocytopenia (OR 1.054, 95% CI 1.002–1.108; *p* = 0.042). MR‐Egger, weighted‐median, simple‐mode, and weighted‐mode estimates were not statistically significant (Table 4).

**Table 4:** Mendelian randomization estimates for the association of rheumatoid arthritis liability with FinnGen D69.6 thrombocytopenia.

Method	OR	95% CI	*p* value
IVW (fixed effect)	1.054	1.002–1.108	0.042
Weighted median	0.951	0.877–1.032	0.232
MR‐Egger	0.952	0.880–1.030	0.225
Simple mode	1.146	0.933–1.407	0.199
Weighted mode	0.976	0.909–1.048	0.507

*Note:* All estimates used 52 harmonized SNPs.

Abbreviations: CI, confidence interval; IVW, inverse variance weighted; MR, Mendelian randomization; OR, odds ratio.

There was no strong evidence of heterogeneity (IVW Q = 63.84, *p* = 0.107; MR‐Egger Q = 52.20, *p* = 0.388). However, the MR‐Egger intercept indicated directional horizontal pleiotropy (intercept = 0.02296, SE = 0.00688; *p* = 0.0016). MR‐PRESSO did not identify a statistically significant global outlier signal (global *p* = 0.0555), and no outliers were detected. Leave‐one‐out estimates remained in the same direction, although removal of 17 SNPs moved the nominal IVW *p* value to ≥ 0.05 (Figure 3). Reverse MR could not be performed because FinnGen D69.6 contained no genome‐wide significant variants at *P* < 5 × 10^−8^.

**Figure 3:**
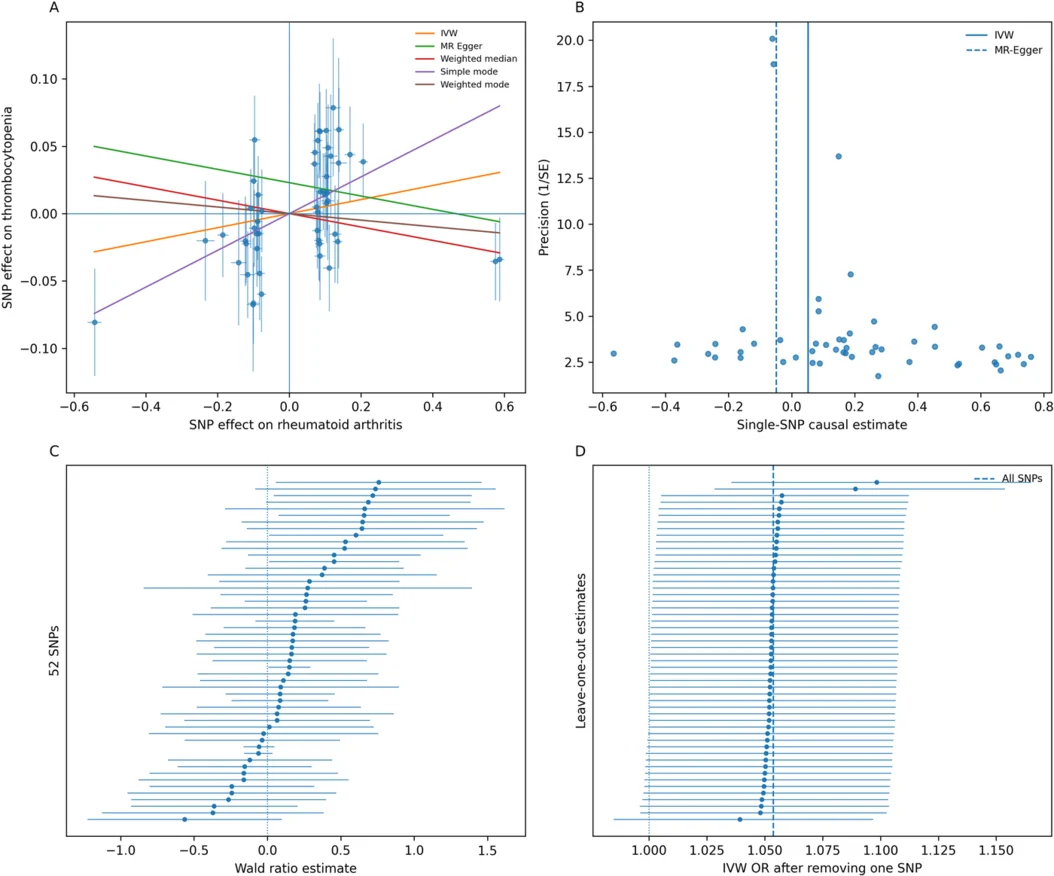
Mendelian randomization analyzes of rheumatoid arthritis liability on FinnGen D69.6 thrombocytopenia. (A) MR scatter plot. (B) Funnel plot. (C) Single‐SNP forest plot. (D) Leave‐one‐out analysis. The fixed‐effect IVW estimate was nominally significant, while the MR‐Egger intercept indicated directional pleiotropy.

### 3.5 Exploratory SMR Target‐Gene Analyzes

For the primary D69.6 outcome, neither JAK1 (SMR *p* = 0.191) nor JAK2 (SMR *p* = 0.062) was statistically significant, and no target in the exploratory panel survived false‐discovery‐rate or Bonferroni correction. For D69.5, IFNG showed a nominal association (SMR *p* = 0.036; HEIDI *p* = 0.943), but this signal did not survive multiple‐testing correction (FDR = 0.335; Bonferroni‐adjusted *p* = 0.393) and was not replicated for D69.6 (Supporting Information Table S4).

## 4 Discussion

Across the observational and genetic analyzes, low platelet counts in RA emerged as clinically relevant. TP < 150 × 10^9^/L was associated with poorer survival after survey‐weighted multivariable adjustment. The revised IVW MR result showed only a nominal association between RA genetic liability and thrombocytopenia, while the complementary estimators and pleiotropy assessment warrant a cautious interpretation.

The two analytic components answer related but distinct questions. The NHANES analyzes describe the clinical and prognostic profile of RA participants with TP, whereas MR addresses whether genetic predisposition to RA is compatible with a directional effect on thrombocytopenia. The two components should therefore not be interpreted as mutually confirmatory evidence of causality.

The primary platelet‐count threshold of < 150 × 10^9^/L was selected because it corresponds to the conventional clinical definition of thrombocytopenia and is consistent with the broad unspecified thrombocytopenia phenotype represented by ICD‐10 D69.6 in the genetic analysis. The stricter < 100 × 10^9^/L threshold was additionally examined as a sensitivity analysis [7, 9, 10, 11]. Results remained directionally consistent, although the estimate was less precise because relatively few participants had platelet counts below 100 × 10^9^/L. Rather than establishing TP as a direct consequence of active RA, our findings suggest that it may help identify participants with a less favorable clinical trajectory.

Albumin and urinary albumin concentration were particularly informative in the fully adjusted RA mortality model. Albumin may integrate nutritional reserve, systemic inflammation, hepatic synthesis, and disease burden, consistent with prior RA studies of albumin‐containing prognostic indices [32, 33, 34, 35, 36, 37]. Albuminuria may indicate endothelial or renal microvascular injury and has been variably linked to vascular abnormalities in RA [38, 39, 40, 41]. These observations are associative and should not be interpreted as evidence that albumin replacement or any renal‐directed intervention would improve survival.

The albumin findings should therefore be viewed as risk‐stratification signals rather than direct therapeutic targets. In routine care, reduced serum albumin or elevated urinary albumin concentration may warrant closer assessment of nutritional status, inflammation, renal involvement, and competing comorbidities in RA patients.

Exploratory SMR analyzes of JAK‐related targets did not provide robust support for a JAK‐centered mechanism. The nominal IFNG signal for D69.5 did not survive multiple‐testing correction and was not replicated for D69.6. Nevertheless, building on previous clinical investigations from our group, baricitinib has shown therapeutic activity in difficult‐to‐treat primary immune thrombocytopenia, first in an open‐label pilot study and subsequently in a randomized phase 2 trial of low‐dose baricitinib plus danazol [42, 43]. These findings suggest that JAK‐mediated immune regulation may remain clinically relevant to immune‐mediated thrombocytopenia, although our present genetic analyzes do not establish this pathway as a mechanism linking RA to thrombocytopenia. Future mechanistic studies are warranted to further investigate the role of JAK‐mediated immune regulation in the development of thrombocytopenia in RA.

Several limitations merit emphasis. The NHANES analysis is observational and may retain residual confounding. RA status was questionnaire based, severe thrombocytopenia was uncommon, and residual confounding remains possible despite adjustment for available demographic, clinical, and medication‐related factors. The association was attenuated after exclusion of participants with current liver conditions, indicating that comorbidity and the resulting change in sample composition may partly contribute to the observed association. In addition, external generalizability requires confirmation, and the MR findings should be interpreted conservatively because the nominal IVW association was not consistently supported by alternative estimators and the MR‐Egger intercept suggested directional pleiotropy.

Although prescription‐medication data were incorporated in sensitivity analyzes, NHANES does not provide complete longitudinal treatment histories, treatment adherence, or comprehensive over‐the‐counter medication exposure. Longitudinal measures of RA disease activity were also unavailable. Therefore, treatment‐related platelet changes and time‐varying inflammatory burden could not be fully disentangled. These limitations mean that the NHANES findings describe prognostic associations rather than treatment effects or a complete causal pathway.

## 5 Conclusions

In this integrated analysis, TP was associated with greater all‐cause mortality in RA, whereas MR did not provide robust evidence that RA genetic liability causally increases TP risk. Lower serum albumin and higher urinary albumin concentration were associated with mortality in the fully adjusted RA model. These findings support closer clinical attention to platelet abnormalities in RA and motivate prospective studies incorporating medication exposure, disease activity, and mechanistic immune data.

## Author Contributions

**Yejun Wu:** conceptualization, methodology, software, data curation, investigation, formal analysis, supervision, funding acquisition, visualization, project administration, resources, writing – original draft, writing – review and editing. **Yuyang Cai:** investigation, software, formal analysis, writing – original draft, data curation. **Anzhen Yang:** validation. **Shuying Zhang:** validation. **Liangyu Xu:** validation. **Yanting Xie:** validation. **Peng Zhao:** conceptualization, methodology, writing – review and editing, visualization, supervision, project administration.

## Ethics Statement

This study used publicly available, de‐identified data from the NHANES database, which was approved by the National Center for Health Statistics Ethics Review Board with all participants providing written informed consent. As this was a secondary analysis of anonymized data, no additional ethical approval was required. The GWAS summary data used in the Mendelian randomization analysis were obtained from studies that had received appropriate ethical approval and informed consent, as detailed in the original publications, and no individual‐level data were used in this study. Hence, ethical approval was not applicable.

## Conflicts of Interest

The authors declare no conflicts of interest.

## Supporting information


**Table S1:** Sensitivity analyses of the association between thrombocytopenia and all‐cause mortality.
**Table S2:** Proportional hazards diagnostics and time‐varying coefficient sensitivity analyses.
**Table S3:** Genetic instruments used in the MR analysis, including per‐SNP F statistics. SNP‐specific R^2^ was not estimated because study‐specific effect‐allele frequencies were unavailable.
**Table S4:** Exploratory SMR analyses of selected JAK‐related inflammatory and downstream signaling targets for D69.6 and D69.5 thrombocytopenia outcomes.

## Data Availability

The data supporting this study are derived from publicly available sources. NHANES data are accessible at https://www.cdc.gov/nchs/nhanes/. European‐ancestry RA GWAS summary statistics (GCST90132223) are available through the NHGRI‐EBI GWAS Catalog. Summary statistics for thrombocytopenia were obtained from FinnGen Release 12 endpoint D3_THROMBOCYTOPENIANAS (ICD‐10 D69.6). Cis‐eQTL data for exploratory SMR analyzes were obtained from the eQTLGen Consortium. The R code used for analysis is available from the corresponding author upon reasonable request.
