# Supplementary material for: Rheumatoid Arthritis and Thrombocytopenia: Causal Evidence and Prognostic Implications From Observational and Mendelian Randomization Analyzes
Source: Immun Inflamm Dis. 2026 Nov 11;14(11):e70551. doi: 10.1002/iid3.70551 (PMC13753078; doi:10.1002/iid3.70551)
Supplement: Supplementary file 1 [file IID3-14-e70551-s001.docx]

**Supplementary Table S1. Sensitivity analyses of the association between thrombocytopenia and all-cause mortality.**

| **Analysis** | **N** | **HR (95% CI)** | ***P* value** |
| --- | --- | --- | --- |
| Primary survey-weighted Model 4 | 1963 | 1.92 (1.17–3.16) | 0.009 |
| BMI time-varying sensitivity (Model 3) | 1963 | 2.32 (1.37–3.95) | 0.002 |
| Urinary albumin time-varying sensitivity (Model 4) | 1963 | 1.91 (1.15–3.15) | 0.012 |
| Stricter threshold: TP <100 ×10^9^/L (Model 4) | 1963 | 3.33 (1.20–9.22) | 0.021 |
| Medication-adjusted Model 4 | 1929 | 1.91 (1.17–3.14) | 0.010 |
| Exclude current liver condition | 1871 | 1.48 (0.87–2.55) | 0.151 |
| Exclude hematologic malignancy history | 1936 | 1.94 (1.17–3.21) | 0.010 |
| Exclude current liver condition and hematologic malignancy history | 1867 | 1.50 (0.88–2.58) | 0.139 |
| Unweighted Model 4 | 1963 | 1.69 (1.16–2.47) | 0.006 |
| Examination-based follow-up time (Model 4) | 1963 | 1.94 (1.18–3.19) | 0.009 |

Abbreviations: HR, hazard ratio; CI, confidence interval; BMI, body mass index; TP, thrombocytopenia.

**Supplementary Table S2. Proportional hazards diagnostics and time-varying coefficient sensitivity analyses.**

**A. Schoenfeld-residual proportional hazards diagnostics**

| **Model** | **Term** | **PH test *P* value** |
| --- | --- | --- |
| Model 3 | Global test | 0.521 |
| Model 3 | Thrombocytopenia | 0.499 |
| Model 3 | Body mass index | 0.044 |
| Model 4 | Global test | 0.170 |
| Model 4 | Thrombocytopenia | 0.365 |
| Model 4 | Natural log-transformed urinary albumin | 0.022 |

**B. Time-varying coefficient sensitivity analyses**

| **Sensitivity model** | **TP HR (95% CI)** | ***P* value** | **Corresponding primary model TP estimate** |
| --- | --- | --- | --- |
| BMI time-varying coefficient in Model 3 | 2.32 (1.37–3.95) | 0.002 | 2.32 (1.37–3.96), *P*=0.002 |
| Urinary albumin time-varying coefficient in Model 4 | 1.91 (1.15–3.15) | 0.012 | 1.92 (1.17–3.16), *P*=0.009 |

Abbreviations: PH, proportional hazards; TP, thrombocytopenia; HR, hazard ratio; CI, confidence interval; BMI, body mass index.

**Supplementary Table S3. Genetic instruments used in the Mendelian randomization analysis, including per-SNP F statistics.**

| **SNP** | **β (RA)** | **SE (RA)** | ***P* (RA)** | **F statistic** |
| --- | --- | --- | --- | --- |
| rs1004095 | 0.5744 | 0.0137 | 0 | 1757.87 |
| rs10173253 | 0.0783 | 0.0138 | 1.324e-8 | 32.19 |
| rs10905284 | -0.0984 | 0.0149 | 3.812e-11 | 43.61 |
| rs10972201 | 0.0994 | 0.0137 | 4.284e-13 | 52.64 |
| rs10985070 | -0.0786 | 0.0131 | 1.922e-9 | 36.00 |
| rs11089637 | 0.111 | 0.0175 | 2.123e-10 | 40.23 |
| rs113532504 | 0.1215 | 0.0215 | 1.606e-8 | 31.94 |
| rs115284761 | -0.1166 | 0.021 | 2.781e-8 | 30.83 |
| rs11810143 | 0.1148 | 0.0195 | 3.632e-9 | 34.66 |
| rs12137270 | 0.0846 | 0.0151 | 2.198e-8 | 31.39 |
| rs12474386 | -0.1 | 0.0129 | 1.026e-14 | 60.09 |
| rs12506688 | 0.1341 | 0.0139 | 3.713e-22 | 93.07 |
| rs137687 | -0.0893 | 0.013 | 7.791e-12 | 47.19 |
| rs16903108 | -0.142 | 0.0212 | 1.958e-11 | 44.86 |
| rs1696466 | 0.0707 | 0.0129 | 3.939e-8 | 30.04 |
| rs17264332 | 0.1684 | 0.015 | 4.143e-29 | 126.04 |
| rs17534670 | -0.0904 | 0.0127 | 9.802e-13 | 50.67 |
| rs2045793 | -0.0971 | 0.0153 | 2.366e-10 | 40.28 |
| rs213196 | -0.2347 | 0.0247 | 1.975e-21 | 90.29 |
| rs2240336 | -0.0856 | 0.0134 | 1.886e-10 | 40.81 |
| rs2409780 | 0.1076 | 0.0161 | 2.233e-11 | 44.67 |
| rs2451279 | 0.0856 | 0.0134 | 1.844e-10 | 40.81 |
| rs2476601 | -0.5439 | 0.0197 | 3.750e-168 | 762.26 |
| rs2561477 | -0.0915 | 0.0139 | 4.955e-11 | 43.33 |
| rs2664035 | 0.0815 | 0.013 | 3.848e-10 | 39.30 |
| rs2793108 | 0.0703 | 0.0129 | 4.620e-8 | 29.70 |
| rs3087243 | -0.1208 | 0.0128 | 2.717e-21 | 89.07 |
| rs3130396 | 0.2057 | 0.0128 | 5.806e-58 | 258.25 |
| rs3184504 | -0.0873 | 0.0135 | 1.042e-10 | 41.82 |
| rs34695944 | 0.1036 | 0.0129 | 1.025e-15 | 64.50 |
| rs4239702 | 0.1036 | 0.0144 | 5.983e-13 | 51.76 |
| rs4584833 | 0.084 | 0.0134 | 3.512e-10 | 39.30 |
| rs4853458 | -0.1236 | 0.0149 | 9.844e-17 | 68.81 |
| rs4916340 | 0.0836 | 0.0148 | 1.516e-8 | 31.91 |
| rs5019428 | 0.0943 | 0.0136 | 4.598e-12 | 48.08 |
| rs548234 | -0.077 | 0.0133 | 7.302e-9 | 33.52 |
| rs5745271 | 0.0749 | 0.0131 | 1.140e-8 | 32.69 |
| rs6429207 | -0.1009 | 0.017 | 2.763e-9 | 35.23 |
| rs6570194 | 0.1369 | 0.0235 | 5.531e-9 | 33.94 |
| rs6659932 | -0.1019 | 0.0176 | 7.192e-9 | 33.52 |
| rs678347 | -0.0835 | 0.0141 | 3.246e-9 | 35.07 |
| rs706778 | 0.1048 | 0.0128 | 3.371e-16 | 67.04 |
| rs7170151 | 0.1022 | 0.0143 | 9.486e-13 | 51.08 |
| rs72928038 | 0.1068 | 0.0184 | 6.277e-9 | 33.69 |
| rs7731626 | -0.186 | 0.0155 | 4.196e-33 | 144.00 |
| rs77509998 | 0.1269 | 0.0169 | 6.840e-14 | 56.38 |
| rs8026898 | 0.1375 | 0.014 | 9.903e-23 | 96.46 |
| rs8133843 | 0.0851 | 0.014 | 1.334e-9 | 36.95 |
| rs9268949 | 0.5861 | 0.0138 | 0 | 1803.79 |
| rs9310852 | 0.0791 | 0.013 | 1.079e-9 | 37.02 |
| rs9603608 | -0.1087 | 0.0137 | 2.315e-15 | 62.95 |
| rs998731 | 0.0771 | 0.0137 | 2.003e-8 | 31.67 |

Abbreviations: RA, rheumatoid arthritis; SNP, single nucleotide polymorphism; SE, standard error.

**Supplementary Table S4. Exploratory SMR analyses of selected JAK-related inflammatory and downstream signaling targets for D69.6 and D69.5 thrombocytopenia outcomes.**

**Panel A. FinnGen D69.6 thrombocytopenia, unspecified**

| **Gene** | **Top SNP** | **β_SMR_** | **SE** | ***P*_SMR_** | ***P*_HEIDI_** | **BH-FDR** | **Bonferroni- adjusted *P*** |
| --- | --- | --- | --- | --- | --- | --- | --- |
| IFNG | rs2870960 | -0.226851 | 0.325446 | 0.4857728 | 0.9267469 | 0.7633572571 | 1 |
| IL1B | rs6542082 | -0.372877 | 0.432053 | 0.388118 | 0.7764007 | 0.7633572571 | 1 |
| TNFRSF1A | rs1800692 | -0.0538327 | 0.114394 | 0.6379341 | 0.07179516 | 0.7796972333 | 1 |
| TNFRSF1B | rs6697733 | 0.282334 | 0.190357 | 0.1380261 | 0.3693232 | 0.6991152667 | 1 |
| IL6 | rs10265117 | 0.287884 | 0.376297 | 0.4442444 | 0.6168649 | 0.7633572571 | 1 |
| JAK1 | rs310233 | 0.61027 | 0.466351 | 0.1906678 | 0.6277986 | 0.6991152667 | 1 |
| JAK2 | rs6476941 | 0.262167 | 0.140591 | 0.06221748 | 0.03771859 | 0.68439228 | 0.68439228 |
| STAT1 | rs6745710 | 0.226204 | 0.465554 | 0.6270517 | 0.3156739 | 0.7796972333 | 1 |
| STAT3 | rs1053004 | 0.0103527 | 0.205731 | 0.9598663 | 0.6116728 | 0.9598663 | 1 |
| STAT5A | rs9909659 | -0.150815 | 0.21519 | 0.4833975 | 0.6568386 | 0.7633572571 | 1 |
| STAT5B | rs185478092 | 0.0675789 | 0.271018 | 0.8030883 | 0.7414808 | 0.88339713 | 1 |

**Panel B. FinnGen D69.5 secondary thrombocytopenia**

| **Gene** | **Top SNP** | **β_SMR_** | **SE** | ***P*_SMR_** | ***P*_HEIDI_** | **BH-FDR** | **Bonferroni- adjusted *P*** |
| --- | --- | --- | --- | --- | --- | --- | --- |
| IFNG | rs2870960 | -1.74526 | 0.830961 | 0.03570274 | 0.943162 | 0.33508673 | 0.39273014 |
| IL1B | rs6542082 | -0.443732 | 1.08048 | 0.6813077 | 0.325564 | 0.92776673 | 1 |
| TNFRSF1A | rs1800692 | -0.0844751 | 0.287499 | 0.76889 | 0.9971401 | 0.92776673 | 1 |
| TNFRSF1B | rs6697733 | -0.0259281 | 0.477836 | 0.9567268 | 0.6367151 | 0.9567268 | 1 |
| IL6 | rs10265117 | -0.610949 | 0.955989 | 0.5227737 | 0.3147763 | 0.92776673 | 1 |
| JAK1 | rs310233 | -1.51256 | 1.16415 | 0.1938474 | 0.5179137 | 0.53308035 | 1 |
| JAK2 | rs6476941 | -0.285123 | 0.352572 | 0.4186915 | 0.6754606 | 0.9211213 | 1 |
| STAT1 | rs6745710 | 2.01251 | 1.19216 | 0.09138729 | 0.6692585 | 0.33508673 | 1 |
| STAT3 | rs1053004 | -0.102022 | 0.516525 | 0.8434243 | 0.7858872 | 0.92776673 | 1 |
| STAT5A | rs9909659 | 0.215506 | 0.541084 | 0.6904191 | 0.2630679 | 0.92776673 | 1 |
| STAT5B | rs185478092 | -1.2233 | 0.690118 | 0.0762952 | 0.4717825 | 0.33508673 | 0.8392472 |

Abbreviation: SMR, summary-data-based Mendelian randomization; HEIDI, heterogeneity in dependent instruments; SNP, single-nucleotide polymorphism; SE, standard error.
